# Short term clinical and echocardiography outcomes of pericardiectomy in constrictive pericarditis

**DOI:** 10.34172/jcvtr.2021.23

**Published:** 2021-04-26

**Authors:** Madhur Kumar, Ajit Padhy, Ridhika Munjal, Anubhav Gupta

**Affiliations:** Department of Cardiothoracic & Vascular Surgery Safdarjung Hospital & Vardhman Mahavir Medical College, New Delhi, India

**Keywords:** Echocardiography, Constrictive Pericarditis, Outcome, Pericardiectomy, NYHA

## Abstract

***Introduction:*** Tuberculous pericarditis continues to be a leading cause of chronic constrictive pericarditis (CCP) in developing countries. Echocardiography plays a key role in the assessment and diagnosis.

***Methods:*** Twelve patients who underwent pericardiectomy for CCP in last 18 months of the study period were subjected to clinical and New York Heart Association (NYHA) functional class assessment along with comprehensive echocardiographic evaluation. The data were compared with their preprocedural status.

***Results:*** Significant reduction was noted in the incidence of inferior vena cava (IVC) congestion(*P* < 0.001) and mean left atrial (LA) size from 43.75 ± 4.43 mm to 31.58 ± 3.03 mm (*P* < 0.001), post pericardiectomy.Respiratory variation of 34.17 ± 8.76 % in the mitral E velocity was significantly reduced to 17 ± 3.69 % (*P* < 0.001) after surgery. Similarly, respiratory variation in tricuspid E velocities showed significant reduction from 62.17 ± 13.16 % to 32.58 ± 4.7 % (*P* < 0.001).Prior to pericardiectomy, medial e’ and lateral e’ mitral annular velocities was 15.5±1.24 cm/sec and13.08 ± 1.08 cm/sec, respectively. Following surgery, the medial e’ and lateral e’ was 12.5±1.17 cm/sec(*P* = 0.001) and 15.42±1.83 (*P* = 0.004), respectively.

***Conclusion:*** Echocardiography provides useful insight in pericardial constriction hemodynamics and worthwhile effects of pericardiectomy.

## Introduction

Chronic constrictive pericarditis (CCP) is characterized by the encasement of the heart by scarred fibrotic pericardium leading to impaired diastolic ventricular filling.^1^ Following tuberculous pericarditis, 30%-60% develop CCP as sequelae.^2^

Multiple studies have highlighted the clinical profile and the importance of imaging for diagnosis and appropriate management of constrictive pericarditis.^3,4^ However, outcome in terms of clinical and echocardiography variables post-pericardiectomy in Indian population is lacking.

Therefore, this study was aimed to assess the clinical characteristics and echocardiographic parameters pre- and post-pericardiectomy.

## Material and Methods

This is a retrospective study of 6 months from July 2019 to December 2019 in the department of cardiothoracic & vascular surgery. The data of all patients who underwent pericardiectomy for chronic constrictive pericarditis within the last 18 months of the study period were accessed. The clinical presentation along with the New York Heart Association (NYHA) functional class and findings of echocardiography, done at least 30 days prior to and 6 months following pericardiectomy were recorded.

**Figure 1:**
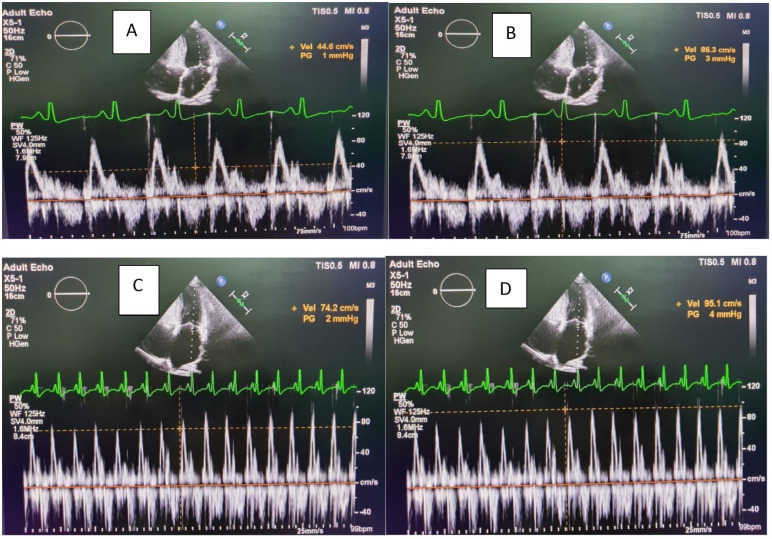
Image of mitral inflow region in a patient showing significant respiratory variation (A,B) pre- operatively as compared to the (C,D) post- operative

**Figure 2:**
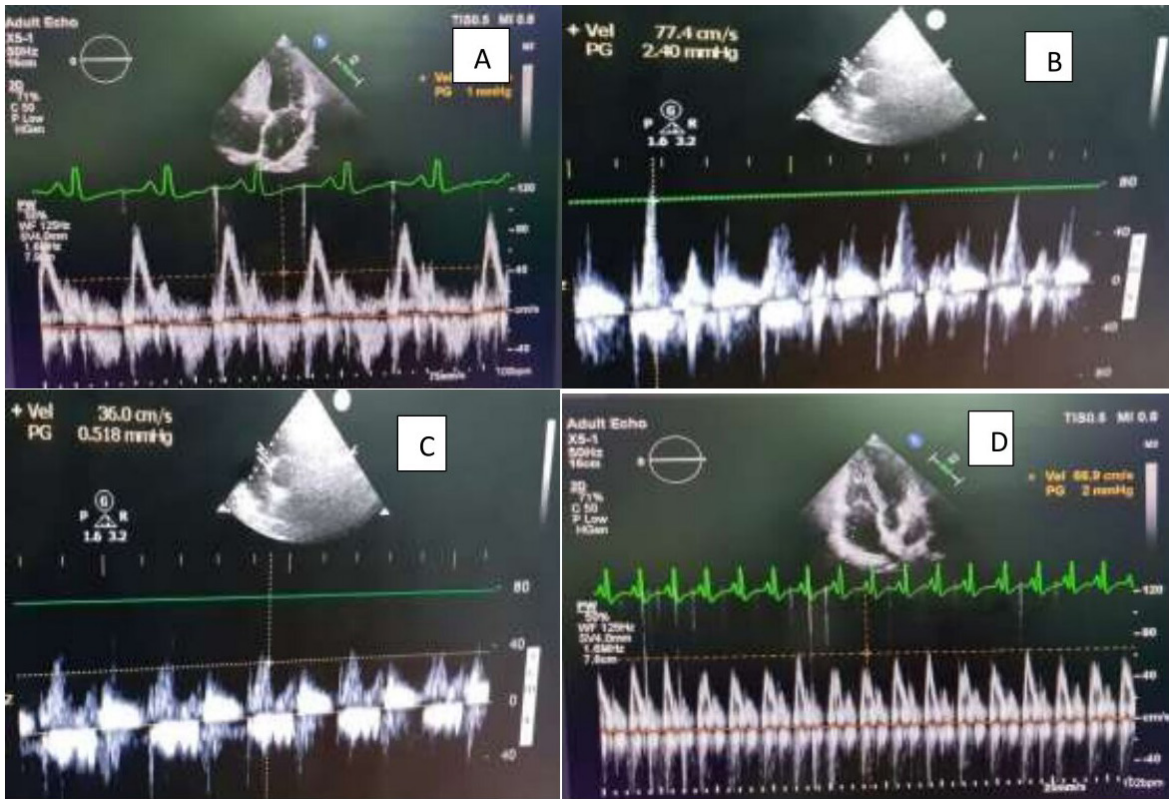
Image of tricuspid inflow region in a patient showing significant respiratory variation (A,B) pre- operatively as compared to the (C,D) post – operative

All patients had comprehensive echocardiographic analysis using PHILIPS EPIQ7C machine with phased array sector probe of 2-4 MHz before and after pericardiectomy. The Left atrium (LA) anteroposterior dimension was measured in parasternal long-axis view. Left ventricle (LV) ejection fraction (EF) was calculated by 2D echocardiography. Mitral and tricuspid valvular regurgitation was assessed semi-quantitatively as grade 1+ to 4+. The dimension, respiro-phasic variation of the inferior vena cava (IVC) and septal bounce was detected on M-mode. Mitral and tricuspid peak velocity of early (E) and late (A) filling were assessed by pulsed wave doppler in apical 4- chamber (A4C) view. The peak E velocities were recorded in both the phases of respiration (assessed clinically) Figure 1 and Figure 2. TDI in A4C in early diastole was used to evaluate peak annular velocities with sample volume of 2–4 mm placed at the septal (medial e’) and lateral (lateral e’) corner along the mitral annular plane.

All patients underwent isolated pericardiectomy without cardiopulmonary bypass. Central venous pressure (CVP) was monitored intraoperatively. All patients were given anti-tubercular treatment for 6 months.

### Statistical analysis

The data analysis was done using Statistical Package for Social Sciences (SPSS) version 21.0.

Categorical variables were presented in number and percentage (%) and continuous variables were presented as mean ± standard deviation (SD) and median. Normality of data was tested by Kolmogorov-Smirnov test. If the normality was rejected then non parametric test was used.

Quantitative variables were compared using paired t test/Wilcoxon signed rank test (when the data sets were not normally distributed) between pre and post echo. Qualitative variables were compared using Chi-Square test/Fisher exact test. A *P* value of < 0.05 was considered statistically significant.

## Results

Amongst the 12 patients studied, 5 (41.6%) were female. In the study population, 7 (58.3%) patients were below 25 years old and 5 (41.6%) were above 25 years old. Dyspnoea, distension of neck veins and ascites were the presenting features in all the patients along with Kussmaul sign. Hepatomegaly and pedal oedema were seen in 8 (66.6%) patients along with atypical chest pain in 2 (16.6%) patients. Pericardial calcification was evident in 20% of patients on chest radiograph and mean pericardial thickness on mediastinal computed tomography was 5.3 ± 1.1 mm.

New York Heart Association (NYHA) functional class improved post-pericardiectomy (Table 1). A comparative analysis of various echocardiography parameters such as IVC congestion, septal bounce, pericardial effusion, tricuspid regurgitation, mitral regurgitation, left atrium size, ejection fraction, mitral E velocity, tricuspid E velocity, mitral annular tissue doppler imaging was performed, pre-pericardiectomy and post- pericardiectomy. (Table 1)

**Table 1:** Comparison of pre- and post- pericardiectomy NYHA functional class and echocardiography parameters (n=12)

**NYHA Functional Class**	**Pre-operative ECHO**	**Post-operative ECHO**	***P*** ** value**
I	0(0%)	8(66.67%)	
II	6(50%)	4(33.33%)	0.009
III	5(41.66%)	0(0%)	
IV	1(8.33%)	0(0%)	
Echocardiography parameters			
IVC congestion n (%)	12(100%)	1(8.33%)	<0.0001
Septal bounce n (%)	12(100%)	0(0%)	<0.0001
Pericardial effusion n (%)	3(25%)	0(0%)	0.217
Tricuspid regurgitation No n (%)	12(100%)	12(100%)	-
Mitral regurgitation No n (%)	12(100%)	12(100%)	-
Left atrium size(mm)			
Mean ± SD	43.75 ± 4.43	31.58 ± 3.03	
Median (IQR)	42(40.75-46.5)	32(29.75-34)	<.0001
Range	39-52	26-37	
Ejection fraction (%)			
Mean ± SD	52.08 ± 2.57	52.08 ± 2.57	
Median (IQR)	50(50-55)	50(50-55)	1
Range	50-55	50-55	
Mitral E velocity (cm/sec)			
Inspiratory			
Mean ± SD	62.92 ± 4.12	71.25 ± 3.57	<0.0001
Median (IQR)	64(59.5-65.5)	72(69.5-74)	
Range	56-69	65-77	
Expiratory			
Mean ± SD	93.58 ± 5.16	86.5 ± 3.37	
Median (IQR)	93.5(89.75-96.5)	86(84-88)	<0.0001
Range	86-104	82-95	
Change (%)			
Mean ± SD	34.17 ± 8.76	17 ± 3.69	<.0001
Median (IQR)	32(28.75-36)	17.5(13-20.25)	
Range	28-60	12-22	
Tricuspid E velocity (cm/sec)			
Inspiratory			
Mean ± SD	82 ± 2.98	72.17 ± 3.38	
Median (IQR)	82(79.75-84.25)	71.5(69.75-75)	<0.0001
Range	78-86	67-78	
Expiratory			
Mean ± SD	38.33 ± 3.55	48.33 ± 2.87	
Median (IQR)	37.5(35.75-40.5)	48(46.75-49.5)	<0.0001
Range	34-46	44-54	
Change (%)			
Mean ± SD	62.17 ± 13.16	32.58 ± 4.7	
Median (IQR)	57(54.5-63.25)	34(29.75-36)	<0.0001
Range	51-90	22-38	
Mitral annular TDI (cm/sec)			
Medial e'			
Mean ± SD	15.5 ± 1.24	12.5 ± 1.17	
Median (IQR)	15.5(14.75-16)	12.5(11.75-13.25)	0.001
Range	14-18	11-14	
Lateral e'		
Mean ± SD	13.08 ± 1.08	15.42 ± 1.83	
Median (IQR)	13(12.75-14)	15.5(14.75-16.25)	0.004
Range	11-15	12-18	

Abbreviations: ECHO, Echocardiography; IVC, inferior vena cava; IQR, interquartile range; NYHA, New York Heart Association; SD, standard deviation; TDI, tissue doppler imaging

‘Annulus reversus’ was observed in 9 (75%) patients and resolved post pericardiectomy universally.

## Discussion

In developing countries, the incidence of tubercular chronic constrictive pericarditis (CCP) ranges from 38%– 83% of cases.^5,6^ Biopsy-proven pericardial tuberculosis was noted in 80% patients.

The sign and symptoms reported in Mayo Clinic were: HF (67%); chest pain (8%); abdominal symptoms (6%); restrictive symptoms (5%); atrial arrhythmias (4%); and severe liver disease (4%). In 6% of the cases, there was low cardiac output, repetitive pleural effusion and syncope.^7^

In our study, all the patients presented with abdominal distension due to ascites, followed by dyspnoea (75%). All the patients had distended neck veins and Kussmaul sign. Pedal oedema was evident in 66.6% patients and atypical chest pain (16.6%).

Two of the patients (4.4%) of constrictive pericarditis were in NYHA class I, 20 (44.4%) in class II, 22 (48.9%) in class III, and 1 (2.2%) in class IV.^8^ In our study, pre-operatively, 50% (6/12) and 41.6% (5/12) patients were NYHA class II &III respectively and 8.3% (1/12) patient was functional class IV. Patient’s NYHA status improved to class I or II postoperatively.

In contrast to western population,^8^ the mean age of patient was 22.83 years old in our study.This could be attributed to high prevalence of tubercular pericarditis in the young population of India.

CCP results in various pathophysiological changes, such as impaired diastolic filling of the ventricles, heightened ventricular interdependence and severance of intracardiac and intrathoracic pressures during respiration.^4,9^

Pericardiectomy through median sternotomy allowed excellent exposure, access and a better possibility of complete resection. Total pericardiectomy involves wide excision of the pericardium between the phrenic nerves and from the great vessels superiorly to the diaphragmatic surface inferiorly.

Usually, 70%–80% patients at 5 years and 40%–50% at 10 years endure favourable cardiovascular outcomes. The operative mortality ranges from 5% to 15%.^10,11^

The central venous pressure (CVP) reduced from a mean of 20.7 ± 2.2 mm Hg preoperatively to 12 ± 3.2 mm Hg closely after surgery. Though intraoperative CVP monitoring was used as a guide for adequate resection, a study by Voila stood against it.^12^

Post pericardiectomy, sudden improvement in clinical and hemodynamic parameters may not be evident and take a gradual course.^13,14^ LA size showed significant (*P* < 0.001) reduction by a mean of 12.1 mm, indicating improved diastolic properties of the heart.

In CCP, ventricular filling is severely restricted and occurs only in early diastole with respiratory variation. This underlines the various hemodynamic changes unique to CCP. ^15^

The doppler echocardiography demonstrates respiratory variation in flow velocity across atrio-ventricular valve to point towards CCP.^16^ This is due to the dissociation of intrathoracic and intracardiac pressures and ventricular interdependence.

The various echocardiographic parameters such as IVC congestion, mean LA size, mitral and tricuspid E velocity and TDI findings significantly improved in a study from central India comprising 23 patients of post-pericardiectomy.^17^ In our study, there was significant respiro-phasic variation in the mitral and tricuspid E velocity in all patients (*P* < 0.001). Tissue Doppler imaging (TDI) is a useful for distinguishing CCP from restrictive cardiomyopathy.^18^

TDI revealed increased e′ velocity of the medial mitral annulus and septal abnormalities corresponding to the “bounce.” The lateral mitral annular e′ is lower than the medial annular e′, termed as *annulus reversus*.^19^ Similar to study by Veress et al,^18^ this phenomenon was present in 75% patients prior to surgery.

The present study is retrospective with small number of patients, and has a short follow-up period. Cardiac catheterization was not done in any of the patients.

## Conclusion

Pericardiectomy offers good functional outcomes. Echocardiography continues to enlighten us about worthwhile effects of pericardiectomy in CCP.

## Acknowledgements

None.

## Ethical approval

This study was approved by Institutional Ethics Committee, Vardhman Mahavir Medical College & Safdarjung Hospital, New Delhi, India. (2020-08/CC-58)

## Competing interest

The authors declare that they have no conflict of interest

## Funding

None.
